# Differences in the Microbiological Profile of Raw and Pasteurized Breastmilk from Hospital and Community-Based Donors at the First Human Milk Bank in Vietnam

**DOI:** 10.3390/nu15020412

**Published:** 2023-01-13

**Authors:** Hoang Thi Tran, Tuan Thanh Nguyen, Oanh Thi Xuan Nguyen, Le Thi Huynh, Le Thi Nguyen, Thao Thi Nguyen, Huong Thi Thanh Le, Debbie Barnett, Gillian Weaver, Roger Mathisen

**Affiliations:** 1 Neonatal Unit and Human Milk Bank, Da Nang Hospital for Women and Children, Da Nang 50506, Vietnam; 2 Department of Pediatrics, School of Medicine and Pharmacy, Da Nang University, Da Nang 50206, Vietnam; 3 Alive & Thrive East Asia Pacific, FHI 360, Hanoi 11022, Vietnam; 4 Microbiology Unit, Da Nang Hospital for Women and Children, Da Nang 50506, Vietnam; 5 School of Biotechnology and Food Technology, Hanoi University of Science and Technology, Hanoi 11615, Vietnam; 6 Milk Bank Scotland, Queen Elizabeth University Hospital, Glasgow G51 4TF, UK; 7 International Milk Banking Specialist and Consultant, Human Milk Foundation, Harpenden AL5 2JQ, UK

**Keywords:** infant feeding, donor human milk, human milk bank, microbiology, newborns, parenteral nutrition, Vietnam

## Abstract

Background: Microbiological quality is one of the key safety standards in human milk bank (HMB) operations. We describe the profiles of bacteria in donor human milk (DHM) before and after the pasteurization of samples collected from breastfeeding women in the hospital and from the community in the first HMB in Vietnam. Methods: Data were collected between February 2017 and January 2022 from an online HMB monitoring system. First, DHM samples were cultured, and the number of colony-forming units (CFU) were counted before (*n* = 708) and after pasteurization (*n* = 1146). The gram-staining method combined with the Vitek 2 Compact system were used to identify types of organisms at the Da Nang Hospital for Women and Children’s Laboratory. Passing criteria for DHM included pre-pasteurization samples had a total colony count <10^5^ CFU/mL and post-pasteurization was <10 CFU/mL. Results: During five years of operation, Da Nang HMB had 491 donors (48.7% were hospital and the rest community donors) who donated an average amount of 14.2 L over 45 days. Of this DHM volume, 84.9% of donor samples passed the pre- and post-pasteurization microbiological tests. DHM from community donors had a higher pass rate (87.8%) compared to that from hospital donors (79.5%). Before pasteurization, 15.4% of DHM samples had a bacteria count <10^3^ CFU/mL, 63.0% had 10^3^-<10^5^ CFU/mL, and 21.6% had ≥10^5^ CFU/mL. Most of the unpasteurized DHM samples (93.0%) had microorganism growth: with one organism (16.4%), two (33.9%), three or more (43.6%). After pasteurization, 17.9% samples had a bacteria count of 1–9 CFU/mL and 7.2% had ≥10 CFU/mL. DHM samples from community donors had a lower bacterial count and number of organisms than those from hospital donors both before and after pasteurization. The highest microorganisms from unpasteurized DHM samples were *Staphylococcus epidermidis* (74.2%), *Acinetobacter* sp. (52.1%), gram-positive bacillus (51.7%), *Staphylococcus coagulase-negative* (15.8%), and *Staphylococcus aureus* (10.5%). Common microorganisms from pasteurized DHM were gram-positive bacillus (21.0%), *Staphylococcus epidermidis* (3.9%), and *Acinetobacter* sp. (0.9%). Samples from the hospital tended to have a higher contamination with those microorganisms than those from community donors. Conclusions: The majority of DHM samples in Da Nang passed microbiological testing criteria. DHM from community donors had higher pass rates than hospital donors. Corrective actions are needed to improve HMB operations and hospital microbiological quality standards, as well as general improvements in water and sanitation.

## 1. Introduction

A human milk bank (HMB) is a service established to recruit and screen donors and store, process, screen, and provide donor human milk (DHM) to infants in need, especially preterm or sick newborns whose mothers are unable to provide any or enough of their own breastmilk [1]. The first HMB in Vietnam was established in Da Nang Hospital for Women and Children in 2017 [2]. In the last five years, the Da Nang HMB has recruited 491 women willing to donate their breastmilk and provided pasteurized donor milk to almost 20,000 infants in need [3]. Following this, four other HMBs have been established in three regions of Vietnam [4].

Human donor milk containing undesirable and/or high levels of bacteria can cause severe infections in recipient infants and lead to a high rate of discarded milk with consequent increased operational costs, as well as waste milk donors’ time and resources [5]. The results of microbiological screening may be influenced by factors ranging from breastmilk expression and handling to administration, transportation, storage conditions, and pasteurization techniques [5,6,7]. Previous studies in developed countries showed that although a wide variety of bacteria are present in DHM, most samples (85–87%) were contaminated with *Staphylococcus* [6]. A study in the US showed that 81% of unpasteurized DHM samples had microorganism growth comprising one (48%), two (25%), three or more (8%) organisms [7]. Pooled DHM from two or more mothers had a higher prevalence of microorganism growth than pooled DHM from one mother [7]. Globally, microbiological profiles of DHM from donors in the hospitals and communities, especially in the context of a lower-middle-income countries, are lacking [5,6,7]. In addition, HMBs around the world follow different procedures for processes such as milk expression, handling, storage, pasteurization, and microbiological testing [5].

To fill the gaps, we conducted this study using extracted data from the HMB monitoring system to describe the profiles of bacteria in donor milk and differences between donor milk collected from hospital donors and community donors, and differences between unpasteurized and pasteurized DHM. Determining the bacterial contents of DHM allows the HMB to devise appropriate strategies to improve the collection, storage, transportation, and processing of DHM [6].

## 2. Methods

### 2.1. Study Data and Donation Selection

Data were collected during the normal operation of the Da Nang HMB from February 2017 to January 2022. The data were collected and uploaded daily to a web-based package that captures all aspects of the HMB processes, such as donor recruitment, screening, and approval, donor milk storage, processing, pasteurizing, bacterial testing, pasteurized donor milk approval, and pasteurized donor milk distribution and usage [3]. For this study, we extracted data directly from the HMB software and removed identification information from all records before data analysis. 

All human milk donations were from registered donors who had completed a written health questionnaire about their medical history and lifestyle, who did not use medications that contraindicated for breastfeeding and agreed to serological screenings for human immunodeficiency virus, hepatitis B virus, hepatitis C virus, and syphilis. The potential donors received training on hand hygiene, cleaning, and maintenance of equipment for breastmilk expression and storage, and the importance of reporting changes in health status. They also provided written informed consent for voluntary donation of breastmilk to the HMB to feed other children in need, as well as for usage of the data for research and quality improvement [2]. The HMB manager approved each donor based on passage of all screening criteria, completion of training, and provision of informed consent. Hospital donors were mothers who had an infant in the Neonatal Intensive Care Unit (NICU), while community donors were mostly mothers of healthy infants in the community with excess milk supply. Mothers who donated from the NICU and continued to donate milk after discharge were also classified as hospital donors. Serological screenings were repeated every 180 days for long-term donors.

### 2.2. Donor Human Milk Collection and Processing

To maintain the safety and quality of its operation, the Da Nang HMB only pools milk from one mother and performs bacteriological tests on pre- and post-pasteurization samples of DHM [2].

The process of milk donation from donors to the HMB and delivery to recipients in the Da Nang Hospital for Women and Children has been published previously [2]. In summary, for donors with infants in the NICU, the raw donor milk was collected and kept in refrigerators in the NICU and transferred to the HMB freezers within 8 h [2]. The milk was stored individually by donor. For donations from the community, expressed breastmilk was stored in freezers or the ice compartment in home refrigerators [2]. The HMB staff collected milk weekly from the donors’ homes and transported it back to the HMB using transport containers with dry ice [2].

Raw donor milk was stored in freezers and pasteurized once the donor had at least 4 L of milk stored. Milk was then defrosted and aliquoted, taking care not to mix donors’ milk together [2]. During our operation, each run of the pasteurizer usually had milk from only one donor, although in the protocol, each run of the pasteurizer allowed milk from up to three donors, which was pooled separately [2]. 

### 2.3. Microbiology Testing 

Microbiological testing and screening of donated human milk samples before and after pasteurization (pre-pasteurized and post-pasteurized milk) are to ensure the safety of donated human milk products [2]. In this HMB, microbiological tests include CFU counting and identifying the microorganisms [2]. Reagents and equipment include level 2 biological safety cabinet, 37 °C CO_2_ 5–10% incubator, sterile pipette, aseptic sample vials, sterile plastic inoculation strips, 5% sheep blood agar, diluent as physiological saline 0.85%. The procedure was carried out at the Microbiology Laboratory in Da Nang Hospital for Women and Children, which met level 2 biosafety standards of the Vietnam Ministry of Health. 

#### 2.3.1. Sample Collection and Transportation

A pre-pasteurization sample was taken after the milk of a single donor was pooled and put into a 2-mL sterile vial, labelled with complete information, and stored in a refrigerator at ≤4 °C at the HMB to wait for the post-pasteurization sample. An unopened container of post-pasteurization milk was randomly selected from the pasteurized batch. HMB staff notified the laboratory at least 30 min prior to transportation to the laboratory so that laboratory staff would be prepared. 

The HMB staff transported the samples using dry-ice boxes to the microbiology laboratory in the same building as the HMB. The microbiology laboratory cultured the samples immediately on arrival.

#### 2.3.2. Inoculation and Incubation

The process was carried out in aseptic conditions for microbiological analysis: washing of hands, sanitizing the working surface of the biological safety cabinet and pipette with 70% alcohol, and turning on UV light in 15 min. The blood agar plate was placed in the 37 °C incubator for 30–60 min to dry the agar surface before inoculation. The milk containers were mixed well before handling. One milliliter of the milk sample was evenly spread over the entire surface area of the sheep blood agar plate, dried in the biosafety cabinet, then incubated at 37 °C in the aerobic CO_2_ incubator for 48 h. The pre-pasteurized milk sample was diluted before inoculation to 10^−2^ and 10^−3^ dilutions. 

To obtain a negative control culture, staff from the microbiology unit used a sterile pipette, aspirated 1.0 mL of the diluent, and spread it evenly over the surface of the blood agar plate. The staff applied a sterile inoculum evenly over the entire surface of the blood agar plate, allowed it to dry in a biosafety cabinet, and incubated it at 37 °C in an aerobic CO_2_ incubator for 48 h. 

#### 2.3.3. Counting of Colony-Forming Unit

If no bacterial growth was observed in the negative control culture, the staff continued reading the microbiological result of milk samples. The total number of colonies on the agar plate was counted using the colony counter or with the naked eye. The results were expressed as colony forming units per 1 mL (CFU/mL). If no bacterial growth was observed, the result was “no bacteria in sample”. If bacterial growth was observed in the negative control, the milk sample was recollected, and the analysis was performed again, noting “negative-positive controls”. Based on the number of colonies counted at the lowest dilution to calculate the number of colonies/mL of sample (CFU/mL), the results were expressed to a × 10^n^ for easy comparison (where 0 < a < 10 CFU/mL).

#### 2.3.4. Identifying Microorganisms

Firstly, staff carried out gram staining of the microorganism for identification. If a member of gram-positive cocci was present, a catalase test was performed. For a positive catalase test (i.e., *Staphylococcus* sp.), coagulase was completed to distinguish between *Staphylococcus aureus* (a positive coagulase test) and others (a negative coagulase test). For a negative catalase test (i.e., *Streptococcus* sp.), Vitek2 Compact (BioMérieux Vitek 2 Compact, USA 2014) was used to identify further.

If a member of gram-positive bacilli was detected, the results were sent back to HMB as gram-positive bacilli. Only for a specific request, Vitek2 Compact was used to distinguish the sub type.

If a member of gram-negative bacilli was present, an oxidase test was performed: negative for *Enterobacteriaceae, Acinetobacter* sp., *Stenotrophomonas* spp., while positive test for *Pseudomonas* spp., *Vibrio* spp., *Aeromonas* spp. Vitek2 Compact was used to distinguish further.

If yeast was suspected, Vitek2 Compact was used to confirm. If mold was suspected, a microscope was used to identify it based on mycelium and spores.

To prepare a sample for Vitek2 Compact, the microorganism suspension was diluted with sterile 0.45% saline into a test tube, reaching a concentration of 0.5–0.63 McFarland for gram-positive and gram-negative bacteria: 2.7–3.3 McFarland for anaerobes and Neisseria and 1.8–2.2 McFarland for fungi. Then, the microorganism suspension and the corresponding identification card were put into the identifier (BioMérieux Vitek 2 Compact, USA 2014). Vitek2 Compact provided the results after about 8–24 h, depending on the type of microorganisms.

#### 2.3.5. Reporting the Results of Microorganisms

The information about the number of CFU/mL and name of bacteria were sent back to the HMB. The HMB staff entered the information into the HMB monitoring system.

### 2.4. Decisions Based on Microbiological Tests

Based on results from the microbiological tests, the HMB manager decided if the pasteurized donor milk should be issued for use. To meet the pre-pasteurization acceptance criteria, all batches containing a total colony count <10^5^ (CFU/mL) were accepted. After pasteurization, <10 CFU/mL was accepted. Donor milk passed the microbiological tests meant both pre-and post-pasteurization tests results were accepted. In addition, pre-pasteurization samples with ≥10^4^ CFU of enterobacteriaceae/mL or ≥10^4^ CFU of *Staphylococcus aureus*/mL, or pre- or post-pasteurization samples having fungi were excluded. 

### 2.5. Data Analysis

We used MS excel (Power Query) to perform data management. Descriptive data analysis was performed using Stata 15.1 (Stata Inc., College Station, TX, USA). 

## 3. Results

### 3.1. General Characteristics

During five years of operation, Da Nang HMB had 491 donors, with an average age of 28 years. More than half of the donors had a college, university education, or higher (56.2%) and obtained a white-collar job, such as office staff, teachers, or health workers (61.1%). The community donors were from Da Nang city and had a higher education profile. There was a similar proportion of donors from the hospital (48.7%) and the community (51.3%) (Table 1). 

The amount of donor milk from the community accounted for two-thirds of the total amount. The average amount of donor milk from each donor was 14.2 L over 45 days: the amount and duration were higher in donors from the community than those from the hospital (Table 1). Of the total donor milk amount, 84.9% passed the pre- and post-pasteurization microbiological tests. The DHM from community donors had a higher pass rate (87.8%) compared to DHM from hospital donors (79.5%) (Table 1). 

### 3.2. The Count of Microorganisms

**Figure 1:**
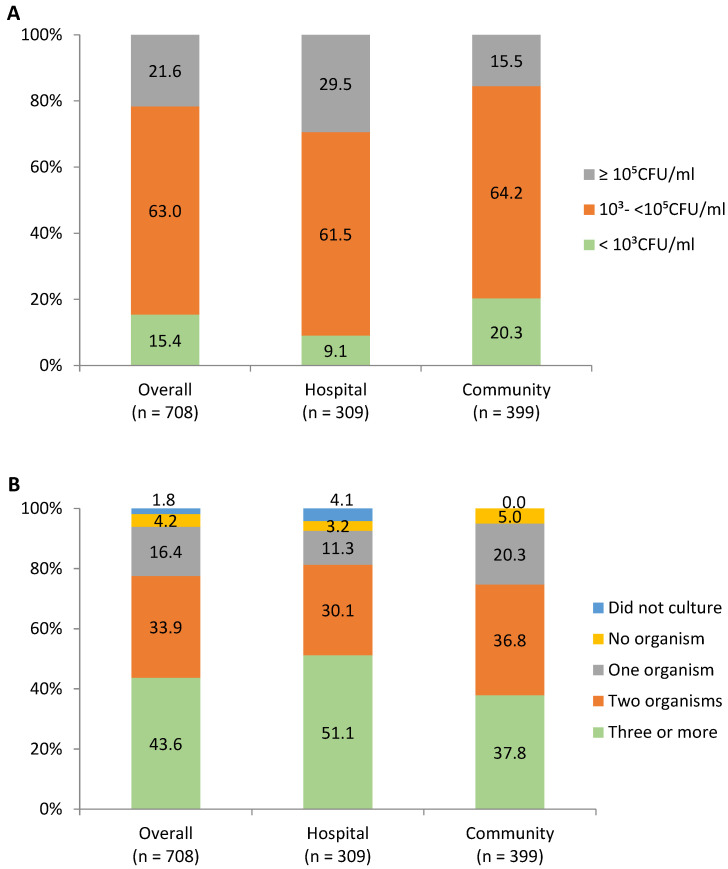
The level of bacteria counts (**A**) and number of microorganisms (**B**) in unpasteurized human donor milk.

For unpasteurized DHM, 15.4% samples had bacteria count <10^3^ CFU/mL, 63.0% had 10^3^-<10^5^ CFU/mL, and 21.6% had ≥10^5^ CFU/mL. Samples from community donors had a lower bacteria count than those from hospital donors, with corresponding values of 20.3% vs. 9.1% for CFU/mL < 10^3^; 64.2% vs. 61.5% for CFU/mL of 10^3^-<10^5^. Most of the unpasteurized DHM samples (93.0%) had a microorganism growth: with one organism (16.4%), two (33.9%), three or more (43.6%). Corresponding percentages for samples from community donors were 20.3%, 36.8%, and 37.8% and from hospital donors were 11.3%, 30.1%, and 51.1% (Figure 1).

**Figure 2:**
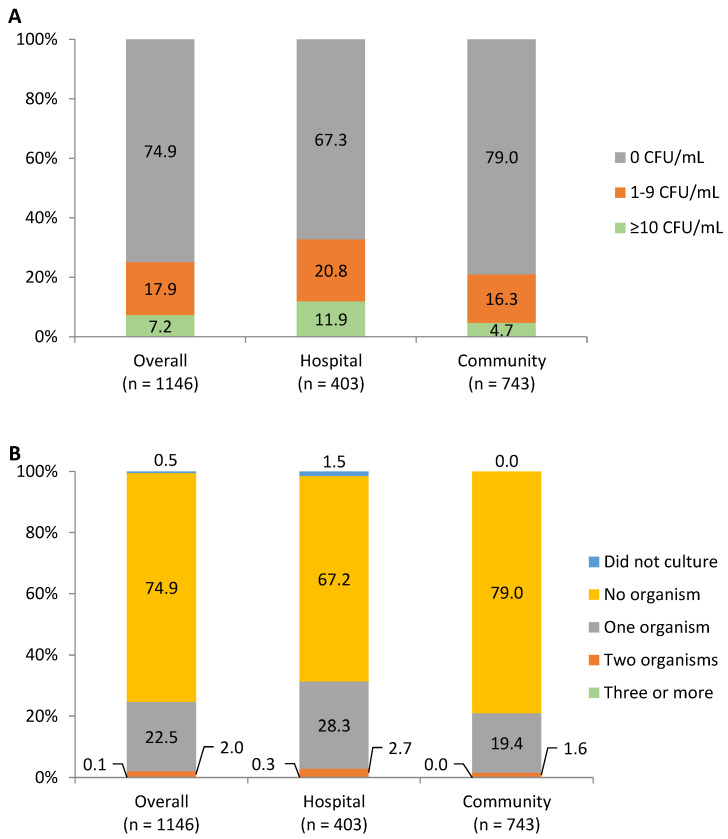
The level of bacteria counts (**A**) and number of microorganisms (**B**) contaminating pasteurized milk.

After pasteurization, 17.9% samples had a bacteria count of 1–9 CFU/mL, and 7.2% had ≥10 CFU/mL. Corresponding percentages for samples from the community were lower than those from the hospital (16.3% vs. 20.8% for 1–9 CFU/mL and 4.7% vs. 11.9 for ≥10 CFU/mL (Figure 2). Most pasteurized samples had no microorganism (74.9% for overall, 67.2% for hospital, and 79.0% for community) or one microorganism (22.5% for overall, 28.3% for hospital, and 19.4% for community) (Figure 2).

### 3.3. Types of Microorganisms

For unpasteurized DHM, the top ten microorganisms were *Staphylococcus epidermidis* (74.2%), *Acinetobacter* sp. (52.1%), gram-positive bacillus (51.7%), *Staphylococcus coagulase-negative* (15.8%), *Staphylococcus aureus* (10.5%), *Pseudomonas* sp. (5.7%), *Enterococcus* sp. (2.3%), *Streptococcus* sp. (2.5%), *Klebsiella* sp. (2.1%), and *E coli* (1.8%) (Table 2). Samples from hospital tended to have a higher contamination by *Acinetobacter* sp., *Staphylococcus coagulase negative*, *Staphylococcus aureus*, *Pseudomonas* sp., and *Pseudomonas aeruginosa* than the samples from the community (Table 2).

For pasteurized DHM, the identified microorganisms were *Staphylococcus epidermidis* (3.9%), *Acinetobacter* sp. (0.9%), gram-positive bacillus (21.0%), *Staphylococcus coagulase-negative* (0.1%), *Staphylococcus aureus* (0.1%), *Pseudomonas* sp. (0.1%), *Candida albicans* (0.2%), and *Candida* spp. (0.1%) (Table 2). Samples from hospital tended to have a higher contamination by *Staphylococcus epidermidis*, *Acinetobacter* sp., and gram-positive bacilli than those from the community (Table 2).

## 4. Discussion

Compared to figures from other Asian HMBs, Da Nang HMB had a higher prevalence of preterm birthing among hospital donors [8,9,10]. This is a special characteristic of Da Nang HMB. Mothers of preterm infants spend at least 20 h per day in kangaroo mother care (KMC) in the hospital, which improves their breastmilk supply and thus supports donation of their surplus breastmilk. As Da Nang Hospital for Women and Children is a center for early essential newborn care (EENC) in Vietnam, almost all spontaneous breathing preterm newborns receive KMC. The average length of stay by each very preterm infant on KMC was at least four weeks [2], which enables mothers to practice breastfeeding and support the HMB. As donor milk from preterm mothers often has a higher protein concentration than that from full-term mothers, this provides more optimal nutrition for recipients who are preterm and sick [11]. In Da Nang HMB, community donors had a higher education level compared to donors from the hospital. A study from Brazil showed that mothers with higher education were more likely to be regular donors [12]. With higher education, donors may have had more opportunities to receive or seek information on donation and were more confident in contacting the HMB directly.

The pre- and post-pasteurization pass rate was 79.0% and 92.5%, which is similar to that from previous studies in China and South Korea [10,13], but lower than that in studies from Australia and Spain [14,15]. In our study, the special findings were higher pass rates from community donors than from hospital donors, which was not widely reported in the literature. We also found that DHM from the community donors had a lower total colony count of bacteria compared to milk from hospital donors. Mothers from the community were typically those who expressed milk for their infants, and when they had a surplus, contacted HMB to donate breastmilk. The mothers had good knowledge and skills related to milk expression and storage. At home, the mothers had their own breast pump and separate fridge or freezer and good hygienic facilities. They also had support from other family members in cleaning and disinfecting equipment. With support from HMB staff, they could easily adapt the protocol to maintain high quality of DHM. In contrast, in the hospital, mothers stored raw milk in a common fridge for donor milk. At the end of a working day, HMB staff collected DHM from different areas and brought it to the HMB to store in the HMB’s freezer. Up to 10 mothers along with fathers or relatives would share one room with the milk donors, so basins and bathrooms were limited, and good hygiene practices for cleaning breast milk expression equipment was not always maintained. 

The bacteria in post-pasteurization tests may have come from operational problems with the pasteurizer and process, high prevalence of bacillus spores, contamination during transportation to the laboratory, or following procedures within the laboratory. As the pasteurizer operation was monitored closely and timing and temperature data saved for monitoring, errors from the pasteurization process were not likely. However, as the containers were submerged during the pasteurization, the water used for pasteurization needed to be controlled. During our operation, we improved cleaning methods for the donor milk bacterial culture biosafety cabinet, decreased transportation time for samples going for testing, used a whole container of pasteurized donor milk for testing in the laboratory instead of taking and transferring small samples, thereby reducing the risk of contamination. These changes combined with better hygiene practices led to improvement in the pass rate after pasteurization. 

There was diversity in donor milk microbiology with the presence of gram-positive and gram-negative bacteria and fungus. The dominant bacteria detected before pasteurization were *Staphylococci*, *Acinetobacter* sp., and gram-positive bacillus. However, it is difficult to assess if these are pathogens as all the donors reported being in good health prior to donation. 

The human milk microbiome has been an interest of researchers in recent decades. Several studies have shown the diversity of bacteria and the traditional concepts of the dominance of lactic acid bacteria in breastmilk may no longer be appropriate. As reported in a Polish study of 139 breastmilk samples, the common bacteria were *Staphylococcus epidermidis*, *Streptococcus mitis/oralist*, and *Staphylococcus aureus*. *Candida Albicans* was also present in one sample [16]. A study of 2890 samples from 448 mothers between 2007 and 2011 in Western Australia showed the common bacteria to be coagulase-negative *Staphylococcus* (85.5%) *Acinetobacter* species (8.1%), and *Staphylococcus aureus* (5%) [6]. A study from Taiwan showed milk samples from healthy mothers having bacterial strains with multiple antibiotic resistance [17]. A study in Italy used polymerase chain reaction to profile DHM microbiota (19 samples) and compared its compositional features with the mother’s own milk microbiota (14 samples) from mothers who delivered prematurely, and found that *Staphylococcus* was the predominant of bacteria in both groups, while DHM also had Staphylococcus and Acinetobacter [18]. The bacteriological colonization profiles of milk from mothers delivering prematurely were similar to those of mothers delivering at term [7].

In donor milk samples from donors in this study, probiotic bacteria such as *Lactobacillus* and *Bifidobacterium* were not detected. Our results are different from studies specifically looking for these species. A study from China using metataxonomic (16S rRNA amplicon analysis) methods found a high proportion of samples presenting Lactobacillus [19]. This may be a result of difficulty in isolation due to fastidious growth and incubation requirements. They may need better and more sensitive techniques to identify microorganisms, such as sequencing analysis or polymerase chain reaction for detection.

The dominance of staphylococci may indicate contamination from maternal skin-associated microbial species during breastfeeding. Meanwhile, the presence of Acinetobacter species and gram-positive bacilli may indicate contamination from breastmilk expression equipment, including breast pumps, circuits, or containers. Furthermore, monitoring air quality is an important mission for the HMB because the contamination may occur during the pooling and aliquoting process, though currently this process has been performed under a laminar flow hood in Da Nang HMB. 

The high prevalence of gram-positive bacillus in Da Nang HMB is a challenge as this is present in more than 50% of milk samples from both community and hospital donors. This bacterium is the most common presenting in more than 20% of samples after pasteurization. Bacillus was the most common cause of milk being discarded in several HMBs globally [20]. To eliminate this, a new operating approach was recommended by colleagues from Italy, including donor education, monitoring of the collection and storage of milk at home, pasteurization process, appropriate hygiene practices, and environmental sanitation. This led to a significant reduction in milk discarded from 13.9% to 5.2% [21]. The Da Nang HMB staff did an initial visit to the donors’ home to check general hygiene and the home freezer before deciding if conditions were suitable for storing donor milk. After that, staff visited weekly or fortnightly to collect donor milk and check the temperature of the freezer. To ensure a consistent approach to the environmental sanitation of the HMB, the cleaning procedures for the pasteurizer, laminar flow hood, and freezers were written and performed as a standard operation. Similarly, surface areas and floors were cleaned routinely.

During five years of operation, the Da Nang HMB has improved practices to improve the proportion of milk that passed both the pre- and post-pasteurization tests [3]. These changes include selecting mothers motivated and committed to milk donation, improving donor education, and close supervision of hospital donors in the KMC unit. The infection control process for mothers’ bathrooms and toilets are performed under our guidelines for infection control, although the parent facility is shared and likely to be overcrowded with many parents. We provided sterilizing units to milk donors along with education on the appropriate timing of breast pump sterilization. We also encouraged donors to use sterilizers integrated with dryers if they could afford them. In the HMB, we used a water filter for water entering the pasteurizer. In addition, only appropriately trained staff in the microbiology laboratory are allowed to perform the microbiological testing using separate tools to test HMB samples. In the microbiology testing area, hand hygiene and environmental hygiene were improved to meet the Da Nang HMB quality standards. 

Our study has limitations, and the microbiological testing in Da Nang used conventional techniques of microbiological culture. Better techniques such as sequencing analysis or polymerase chain reaction may be needed in the future for detecting microbiological profiles.

## 5. Conclusions

The majority of DHM in Da Nang passed microbiological testing, and more DHM passed from community donors than that from hospital donors. Microbiological profiles of donor milk from the first human milk bank in Vietnam showed a diversity of bacteria. While hospital donors provided preterm breastmilk to our HMB, the higher rate of bacterial contamination in these donors as compared to donor milk from the community requires close monitoring and support for improvement. Corrective actions are needed to improve HMB operations and hospital quality standards, as well as general improvement in water and sanitation. 

## Figures and Tables

**Table 1:** General characteristics of human milk donors and donor milk.

Characteristics	Total (*n* = 491)	Hospital (*n* = 239)	Community (*n* = 252)
Donor characteristics			
Donor age, Mean ± SD	28.5 ± 4.1	28.2 ± 4.6	28.8 ± 3.7
One child, *n* (%)	299 (60.9)	136 (56.9)	163 (64.7)
Preterm births, *n* (%)	202 (41.1)	182 (76.2)	20 (7.9)
Cesarean sections, *n* (%)	243 (49.5)	98 (41.0)	145 (57.5)
Donors from Da Nang, *n* (%)	348 (70.9)	110 (46.0)	238 (94.4)
Education level			
College, university, or higher, *n* (%)	276 (56.2)	89 (37.2)	187 (74.2)
Diploma (2–3 years after high school), *n* (%)	90 (18.3)	55 (23.0)	35 (13.9)
Up to high school, *n* (%)	125 (25.5)	95 (39.8)	30 (11.9)
Occupation			
Office staff, *n* (%)	194 (39.5)	70 (29.3)	124 (49.2)
Teachers, *n* (%)	47 (9.6)	24 (10.0)	23 (9.1)
Health workers, *n* (%)	59 (12.0)	16 (6.7)	43 (17.1)
Factory workers, *n* (%)	32 (6.5)	22 (9.2)	10 (4.0)
Sale or small traders, *n* (%)	45 (9.2)	29 (12.1)	16 (6.3)
Housewife, *n* (%)	89 (18.1)	59 (24.7)	30 (11.9)
Other (farmer, self-employed service), *n* (%)	25 (4.9)	19 (8.0)	6 (1.2)
Human donor milk and pasteurization			
Amount of donor milk (L)	9504.9	3372.1	6132.8
Amount of donor milk passed pre- and post- pasteurization test, L (%)	8069.7 (84.9)	2682.3 (79.5)	5387.5 (87.9)
Number of pasteurizations	1244	478 (38.4)	766 (61.6)
Number of pre-pasteurization samples tested	708	317	391
Number of post-pasteurization samples tested	1144	421	723
Passed pre-pasteurization test, *n *(%)	559 (79.0)	226 (71.3)	333 (85.2)
Passed post-pasteurization test, *n* (%)	1058 (92.5)	373 (88.6)	685 (94.7)
Average days of donation, median (IQR)	45 (24–96)	30 (20–61)	70 (29–116)
Donors passed all microbiology tests, *n* (%)	321 (65.4)	139 (58.2)	182 (72.2)
Amount of milk per donor, median (IQR) L	14.2 (7.4–25.2)	8.4 (5.0–7.0)	17.5 (8.5–26.3)
Number of pasteurizations per donor, mean (SD)	2.53 ± 2.49	2 ± 2.02	3.04 ± 2.78
Number passed per donor, mean (SD)	2.12 ± 2.49	1.51 ± 1.98	2.70 ± 2.79

**Table 2:** Types of organisms before and after pasteurization ^1^.

	Pre-Pasteurization			Post-Pasteurization		
	Overall (*n* = 708)	Hospital (*n* = 309)	Comm. (*n* = 399)	Overall (*n* = 1146)	Hospital (*n* = 403)	Comm. (*n* = 743)
*Staphylococcus epidermidis*	525 (74.2)	224 (72.5)	301 (75.4)	45 (3.9)	29 (7.2)	16 (2.2)
*Acinetobacter* sp.	369 (52.1)	189 (61.2)	180 (45.1)	10 (0.9)	9 (2.2)	1 (0.1)
*Gram-positive bacilli*	366 (51.7)	156 (50.5)	210 (52.6)	241 (21.0)	95 (23.6)	146 (19.7)
*Staphylococcus coagulase negative*	112 (15.8)	55 (17.8)	57 (14.3)	1 (0.1)	0	1 (0.1)
*Staphylococcus aureus*	74 (10.5)	41 (13.3)	33 (8.3)	1 (0.1)	1 (0.3)	0
*Pseudomonas* sp.	64 (9.0)	35 (11.3)	29 (7.3)	1 (0.1)	1 (0.3)	0
*Pseudomonas aeruginosa*	40 (5.7)	28 (12.5)	12 (3.0)	1 (0.1)	1 (0.3)	0
*Enterococcus* sp.	16 (2.3)	8 (2.6)	8 (2.0)	0	0	0
*Streptococcus* sp.	18 (2.5)	7 (2.3)	11 (2.8)	0	0	0
*Klebsiella* sp.	15 (2.1)	7 (2.3)	8 (2.0)	0	0	0
*Ecoli*	13 (1.8)	7 (2.3)	6 (1.5)	0	0	0
*Serratia* sp.	12 (1.7)	5 (1.6)	7 (1.8)	0	0	0
*Candida albicans*	9 (1.3)	3 (1.0)	6 (1.5)	2 (0.2)	1 (0.3)	1 (0.1)
*Pseudomonas putida*	2 (0.3)	2 (0.7)	0	0	0	0
*Stenomatophia*	2 (0.3)	1 (0.3)	1 (0.3)	0	0	0
*Candida* spp.	3 (0.4)	1 (0.3)	2 (0.5)	1 (0.1)	1 (0.3)	0
*Enterobacter* sp.	2 (0.3)	0	2 (0.5)	0	0	0
*Pantoea* sp.	1 (0.1)	0	1 (0.3)	0	0	0
*Klebsiella pneumonia*	1 (0.1)	0	1 (0.3)	0	0	0
*Mold*	1 (0.1)	0	1 (0.3)	0	0	0
*Stenomatophia*	2 (0.3)	1 (0.3)	1 (0.3)	0	0	0
*Stenotrophoonas maltophilia*	1 (0.1)	0	1 (0.3)	0	0	0
*Yeast*	1 (0.1)	0	1 (0.3)	0	0	0
*Proteus* spp.	1 (0.1)	0	1 (0.3)	0	0	0

^1^ Data were presented as number (column percentage); Comm.: Community.

## Data Availability

Requests for data may be directed to the corresponding author and are subject to institutional data use agreements.

## Abbreviations

CFU: colony-forming units; DHM: donor human milk; HMB: human milk bank; KMC: kangaroo mother care; NICU: neonatal intensive care unit.

## References

[1] Tran H.T., Nguyen T.T., Mathisen R. (2020). The use of human donor milk. BMJ.

[2] Mansen K., Nguyen T.T., Nguyen N.Q., Do C.T., Tran H.T., Nguyen N.T., Mathisen R., Nguyen V.D., Ngo Y.T.K., Israel-Ballard K. (2020). Strengthening Newborn Nutrition Through Establishment of the First Human Milk Bank in Vietnam. J. Hum. Lact..

[3] Tran H.T., Nguyen T.T., Barnett D., Weaver G., Nguyen O.T.X., Van Ngo Q., Le H.T.T., Huynh L.T., Do C.T., Mathisen R. (2021). Trends and Dynamics in the First Four Years of Operation of the First Human Milk Bank in Vietnam. Nutrients.

[4] Human Milk Bank Global Map. https://public.tableau.com/app/profile/human.milk.bank.global.map/viz/HumanMilkBankGlobalMap_0/HumanMilkBankGlobalMap.

[5] PATH (2019). Strengthening Human Milk Banking: A Resource Toolkit for Establishing and Integrating Human Milk Bank Programs—A Global Implementation Framework.

[6] Almutawif Y., Hartmann B., Lloyd M., Erber W., Geddes D. (2017). A retrospective audit of bacterial culture results of donated human milk in Perth, Western Australia. Early Hum. Dev..

[7] Landers S., Updegrove K. (2010). Bacteriological screening of donor human milk before and after Holder pasteurization. Breastfeed. Med..

[8] Chang F.Y., Cheng S.W., Wu T.Z., Fang L.J. (2013). Characteristics of the first human milk bank in Taiwan. Pediatr. Neonatol..

[9] Xiaoshan H., Xue C., Jun Z., Feng L., Xiaohui C., Zhangbin Y., Shuping H. (2022). Eight-year operation status and data analysis of the first human milk bank in East China. Int. Breastfeed. J..

[10] Liu X.H., Han S.P., Wei Q.F., Zheng F.Y., Zhang T., Chen H.M., Mao M. (2019). The data and characteristics of the human milk banks in mainland China. World J. Pediatr..

[11] Gidrewicz D.A., Fenton T.R. (2014). A systematic review and meta-analysis of the nutrient content of preterm and term breast milk. BMC Pediatr..

[12] Pimenteira Thomaz C.A., Maia Loureiro L.V., da Silva Oliveira T., de Mendonça Furtado Montenegro N.C., Dantas Almeida Júnior E., Fernando Rodrigues Soriano C., Calado Cavalcante J. (2008). The human milk donation experience: Motives, influencing factors, and regular donation. J. Hum. Lact..

[13] Jang H.L., Cho J.Y., Kim M.J., Kim E.J., Park E.Y., Park S.A., Kim I.Y., Choi Y.S., Bae C.W., Chung S.H. (2016). The Experience of Human Milk Banking for 8 Years: Korean Perspective. J. Korean Med. Sci..

[14] Clifford V., Klein L.D., Sulfaro C., Karalis T., Hoad V., Gosbell I., Pink J. (2020). What are Optimal Bacteriological Screening Test Cut-Offs for Pasteurized Donor Human Milk Intended for Feeding Preterm Infants?. J. Hum. Lact..

[15] Padín Fontán M., Martín-Forero Maestre M., Rodríguez Otero I., Durán Fernández-Feijoo C., Suárez Albo M., Concheiro Guisán A. (2022). Influence of donor profile on pre and post-pasteurization bacteriology of donated human milk. Nutr. Hosp..

[16] Strom K., Jarzynka S., Minkiewicz-Zochniak A., Barbarska O., Olędzka G., Wesolowska A. (2022). Microbiological Quality of Milk Donated to the Regional Human Milk Bank in Warsaw in the First Four Years of Activity. Healthcare.

[17] Asbury M.R., Butcher J., Copeland J.K., Unger S., Bando N., Comelli E.M., Forte V., Kiss A., LeMay-Nedjelski L., Sherman P.M. (2020). Mothers of Preterm Infants Have Individualized Breast Milk Microbiota that Changes Temporally Based on Maternal Characteristics. Cell Host Microbe.

[18] Beghetti I., Barone M., De Fazio L., Laderchi E., Biagi E., Turroni S., Brigidi P., Pession A., Corvaglia L., Aceti A. (2022). A Pilot Study on Donor Human Milk Microbiota: A Comparison with Preterm Human Milk Microbiota and the Effect of Pasteurization. Nutrients.

[19] Zhang X., Mushajiang S., Luo B., Tian F., Ni Y., Yan W. (2020). The Composition and Concordance of Lactobacillus Populations of Infant Gut and the Corresponding Breast-Milk and Maternal Gut. Front. Microbiol..

[20] Adjidé C.C., Léké A., Mullié C. (2022). Bacillus cereus contamination of pasteurized human milk donations: Frequency, origin, seasonal distribution, molecular typing of strains and proposed corrective/preventive actions. J. Matern. Fetal Neonatal Med..

[21] Mallardi D., Piemontese P., Liotto N., Colombo R.M., Dodaro A., Schiavello A., Tabasso C., Plevani L., Bezze E., Menis C. (2022). New Operating Approach to Limit Bacillus Cereus Contamination of Donor Human Milk. J. Hum. Lact..

